# The Mutational Landscape of *PTK7* in Congenital Scoliosis and Adolescent Idiopathic Scoliosis

**DOI:** 10.3390/genes12111791

**Published:** 2021-11-12

**Authors:** Zhe Su, Yang Yang, Shengru Wang, Sen Zhao, Hengqiang Zhao, Xiaoxin Li, Yuchen Niu, Deciphering Disorders Involving Scoliosis and COmorbidities (DISCO) Study Group, Guixing Qiu, Zhihong Wu, Nan Wu, Terry Jianguo Zhang

**Affiliations:** 1 State Key Laboratory of Complex Severe and Rare Diseases, Department of Orthopedic Surgery, Peking Union Medical College Hospital, Peking Union Medical College and Chinese Academy of Medical Sciences, Beijing 100730, China; suesu0092@126.com (Z.S.); kaido137@163.com (Y.Y.); wangshengru@foxmail.com (S.W.); zhaosen830@163.com (S.Z.); zhaohq921@gmail.com (H.Z.); qiuguixingpumch@126.com (G.Q.); dr.wunan@pumch.cn (N.W.); 2 Graduate School, Peking Union Medical College, Beijing 100005, China; 3 Beijing Key Laboratory for Genetic Research of Skeletal Deformity, Beijing 100730, China; ldx217@yeah.net (X.L.); nhtniuyuchen@126.com (Y.N.); wuzh3000@126.com (Z.W.); 4 Key Laboratory of Big Data for Spinal Deformities, Chinese Academy of Medical Sciences, Beijing 100730, China; 5 Medical Research Center, State Key Laboratory of Complex Severe and Rare Diseases, Peking Union Medical College Hospital, Peking Union Medical College and Chinese Academy of Medical Sciences, Beijing 100730, China

**Keywords:** protein tyrosine kinase 7 (*PTK7*), congenital scoliosis, adolescent idiopathic scoliosis, whole exome sequencing

## Abstract

Depletion of *ptk7* is associated with both congenital scoliosis (CS) and adolescent idiopathic scoliosis (AIS) in zebrafish models. However, only one human variant of *PTK7* has been reported previously in a patient with AIS. In this study, we systemically investigated the variant landscape of *PTK7* in 583 patients with CS and 302 patients with AIS from the Deciphering Disorders Involving Scoliosis and COmorbidities (DISCO) study. We identified a total of four rare variants in CS and four variants in AIS, including one protein truncating variant (c.464_465delAC) in a patient with CS. We then explored the effects of these variants on protein expression and sub-cellular location. We confirmed that the c.464_465delAC variant causes loss-of-function (LoF) of *PTK7*. In addition, the c.353C>T and c.2290G>A variants identified in two patients with AIS led to reduced protein expression of PTK7 as compared to that of the wild type. In conclusion, LoF and hypomorphic variants are associated with CS and AIS, respectively.

## 1. Introduction

Protein tyrosine kinase (*PTK7*), also known as colon carcinomakinase-4 (*CCK-4*), is an evolutionarily conserved atypical receptor tyrosine kinase. The PTK7 protein consists of seven extracellular immunoglobulin (Ig)-like domains, one transmembrane domain and one catalytically inactive kinase domain [1]. *PTK7* plays an important role in vertebrate canonical and non-canonical Wnt (planar cell polarity, PCP), Semaphorin/Plexin and vascular endothelial growth factor (VEGF) signaling pathways [2,3,4,5]. These signaling pathways are important for embryonic developmental processes, including tissue specification, axial morphogenesis, formation of the cardiovascular, endocrine and immune systems, and regulation of neural crest migration and tumorigenesis [6,7,8,9,10].

Zebrafish models depleted of *ptk7* presented various spinal curve phenotypes. Maternal zygotic *ptk7* (MZ*ptk7*) and zygotic *ptk7* (Z*ptk7*) mutant zebrafish develop spinal curvatures that model congenital scoliosis (CS) and adolescent idiopathic scoliosis (AIS), respectively, due to differential timing of ptk7 loss-of-function [2,11]. Meanwhile, a novel sequence variant *PTK7^P545A^* has been reported in a patient with AIS, but without further in vitro investigation [11]. The association between human *PTK7* variants and scoliotic phenotypes continue to be understudied.

In this study, we analyzed variants in *PTK7* identified in a mixed cohort of patients with congenital scoliosis and adolescent idiopathic scoliosis, then performed in vitro experiments to determine the effects of these variants on protein expression and sub-cellular location.

## 2. Materials and Methods

### 2.1. Human Subjects

A total of 885 Han Chinese individuals who received a diagnosis of congenital scoliosis (CS, *n* = 583) and adolescent idiopathic scoliosis (AIS, *n* = 302) were recruited between 2009 and 2018 at Peking Union Medical College Hospital (PUMCH) for the Deciphering disorders Involving Scoliosis and COmorbidities (DISCO, http://discostudy.org/, accessed on 1 November 2021) project. Clinical manifestations, physical examination results, and detailed medical histories were obtained with the patients’ informed consent. Clinical diagnoses were confirmed by radiology imaging, including X-ray and computed tomography (CT). The criteria for the diagnosis of congenital scoliosis and adolescent idiopathic scoliosis were as follow: congenital scoliosis was caused by vertebral defects, and may be associated with rib anomalies, while idiopathic scoliosis was diagnosed by spinal curvature exceeding 10° on a plain antero-posterior X-ray image, with no other identifiable underlying disease. For a diagnosis of adolescent idiopathic scoliosis, patients were required to have an onset age of 10–18 years old. All radiographic evaluations were conducted by trained spine surgeons, while clinical reviews were performed by alternate observers blinded to the radiographic assessment. Patients with a prior molecular diagnosis such as a disease-causing genetic variant were excluded. Blood was obtained from all subjects and whole-exome sequencing (WES) was performed.

Written informed consent for both clinical data and the genetic exome sequencing was obtained from each participant prior to study participation. This study was approved by the Department of Scientific Research and Ethics Committee of PUMCH in China.

### 2.2. Bioinformatic Analysis and Variant Interpretation

Two DNA extraction and purification kits, Red Blood Cell (RBC) Lysis Buffer (R1010, Solarbio) and Circulating Nucleic Acid Kit (55114, Qiagen), were used in accordance with the manufacturers’ protocols. Approximately 4 mL of peripheral blood was transferred to an Eppendorf safe lock tube after sufficient centrifugation. 10 mL RBC lysis solution was added to each centrifuge tube for efficient lysis. 4.5 mL cell lysis solution and 250 µL proteinase K solution were added to each tube and placed at 56.5 °C constant temperature shaker digestion overnight. 1.5 mL protein precipitation solution was added to each tube and allowed to incubate for 10 min at −20 °C. After centrifugation, the supernatant was taken, and 7 mL precooled isopropanol was added into the supernatant until floccule was precipitated. Finally, 1 mL of 75% ethanol was used to wash the DNA pellet after inverting the tube several times, followed by centrifugation at 17,000× *g* for 10 min. The quality and quantity of the DNA was evaluated using a spectrophotometer (NanoPhotometer Pearl, Denville Scientific, Inc., Holliston, MA, USA) and fluorometer (Qubit^®^ dsDNA High Sensitivity and dsDNA Broad Range assay, Life Technologies Corporation, Waltham, MA, USA). DNA samples were prepared in Illumina libraries and then underwent whole-exome capture with the SureSelect Human All Exon V6 + UTR r2 core design (91 Mb, Agilent, Santa Clara, CA, USA), followed by sequencing on the Illumina HiSeq 4000 platform in 150-bp paired-end reads mode (Illumina, San Diego, CA, USA). WES data processing was performed with the Peking Union Medical College Hospital Pipeline (PUMP) based on the reference genome GRCh37-v1.6 [12,13]. Combined Annotation Dependent Depletion (CADD PHRED-score) [14] and Polyphen-2 [15] were used to predict the pathogenicity of candidate variants. Genotype was filtered for read-depth (DP > 10×), genotype quality (GQ > 20), quality by depth (QD < 2), strand odds ratio (SOR > 9), and allele balance (AB > 0.25). The populational frequency of each QC-passed variant was obtained from the public population databases, including the 1000 Genomes Project, the Exome Sequencing Project [16], the Genome Aggregation Database (gnomAD) [17], and the in-house database of DISCO (Deciphering disorders Involving Scoliosis and COmorbidities, http://discostudy.org/, ≈8000 exomes/genomes, accessed on 1 November 2021) study. Rare variants (minor allele frequency < 0.001) were retained for further filtering. From these rare variants, we included the protein-altering or splice-region variants for subsequent analysis. Potential spicing variants were predicted using SpliceAI [18].

Candidate variants of *PTK7* were selected based on the following criteria:

(1) Predicted to alter the protein sequence;
(2) Either absent or with a low frequency (<0.001) from the public database mentioned above.
(3) The missense variants have a CADD score ≥15.

### 2.3. Site-Directed Mutagenesis

pcDNA3.1+ with C-terminal flag-tagged wild type (WT) and variant *PTK7* cDNA (NM_152881.3) plasmids were acquired from Beijing Hitrobio Biotechnology (Beijing, China). The variant constructs were sequenced on both strands to verify nucleotide changes.

### 2.4. Cell Culture and Transfection Assay

HEK293T cells were cultured in Dulbecco’s Modified Eagle’s medium (DMEM, Invitrogen, Carlsbad, CA, USA) supplemented with 10% fetal bovine serum (Gibco, Waltham, MA, USA), penicillin (50 U/mL), and streptomycin (50 μg/mL) in six-well plates. HEK293T cells were transfected with full-length WT or variant *PTK7* constructs using Lipofectamine 3000 (Invitrogen).

### 2.5. Western Blot Assay

After a 48 h transfection, HEK293T cells were harvested in RIPA lysis buffer (Solarbio, Beijing, China) and whole-cell lysate was resolved on gels under reducing conditions, transferred to a Nitrocellulose Transfer (NC) membrane, blocked with non-fat milk for 30 min at room temperature, and probed with primary antibodies: mouse anti-DDK(FLAG) antibody (1:1000, ZSGB-BIO, TA-05) and mouse anti-β-actin antibody (1:5000, Proteintech, 66009-1-Ig) over-night at 4 °C, and then with a goat anti-mouse horseradish peroxidase-conjugated secondary antibody (1:5000, ZSGB-BIO, ZB-2305). Immunoreactivity was visualized using Western Lighting chemiluminescence reagent (Beyotime, Shanghai, China). All Western blotting experiments were repeated three times.

### 2.6. Immuno-Fluorescence

Cells were grown on 35 mm glass bottom cell culture dishes for 48 h after transfection, washed three times, fixed in 4% paraformaldehyde, permeabilized with 0.1% Triton X-100, and incubated with primary anti-DDDDK-tag mouse antibody (1:1000, MBL, M185-3L) at 4 °C overnight. Cells were incubated and stained with secondary antibody Alexa FlourTM 488 goat anti-mouse IgG (1:2000, Invitrogen, 1911843), then covered with DAPI (Solarbio, Beijing, China).

### 2.7. Statistical Analysis

The overall protein expression levels were normalized to WT (set as 1.0) and mean values of variant versus WT from all three experiments were compared using unpaired *t*-test. All charts are drawn and analyzed using GraphPad Prism 8 and *p* < 0.05 was considered significant for all analyses.

## 3. Results

**Figure 1:**
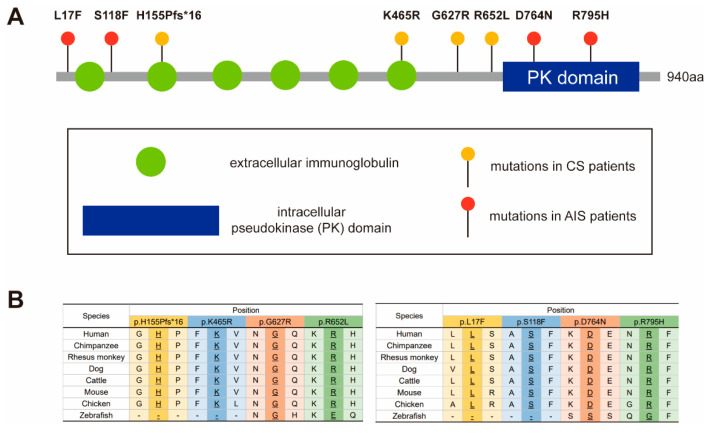
Mapping and conservation analysis of the identified variants. (**A**) Mapping of eight *PTK7* variants revealed that p.L17F, p.S118F, p.H155Pfs*16 and p.K465R are located in the extracellular region of PTK7 protein. p.D764N and p.R795H are located in the intracellular pseudokinase domain. (**B**) The conservation of variants in human and other vertebrate species.

A total of 885 genomes from patients with scoliosis were sequenced and eight *PTK7* variants in nine patients were found. The mean age of the included nine patients with variants was 11.11 ± 5.51 years. In five CS patients, the mean Cobb angle of the coronal plane was 59.94° ± 25.85°. Among them, three patients displayed kyphosis with a mean angle of 50.53° ± 8.05°. The mean Cobb angle of structural curve in four AIS patients was 48.95° ± 4.77°. In the CS group (*n* = 583), four possibly deleterious variants were revealed in five patients, including one frameshift variant and three missense variants (c.464_465delAC, c.1394A>G, c.1879G>A, c. 1955G>T) (Table 1). One of the missense variants (c.1955G>T) was identified in two patients. In the AIS group (*n* = 302), four deleterious missense variants (c.49C>T, c.353C>T, c.2290G>A, c.2384G>A) were identified (Figure 1A, Table 2). No peripheral blood samples from the patients’ families were obtained, and no similar family history of spinal deformity was found after follow-up.

### 3.1. Variant and Phenotypic Characteristics

**Figure 2:**
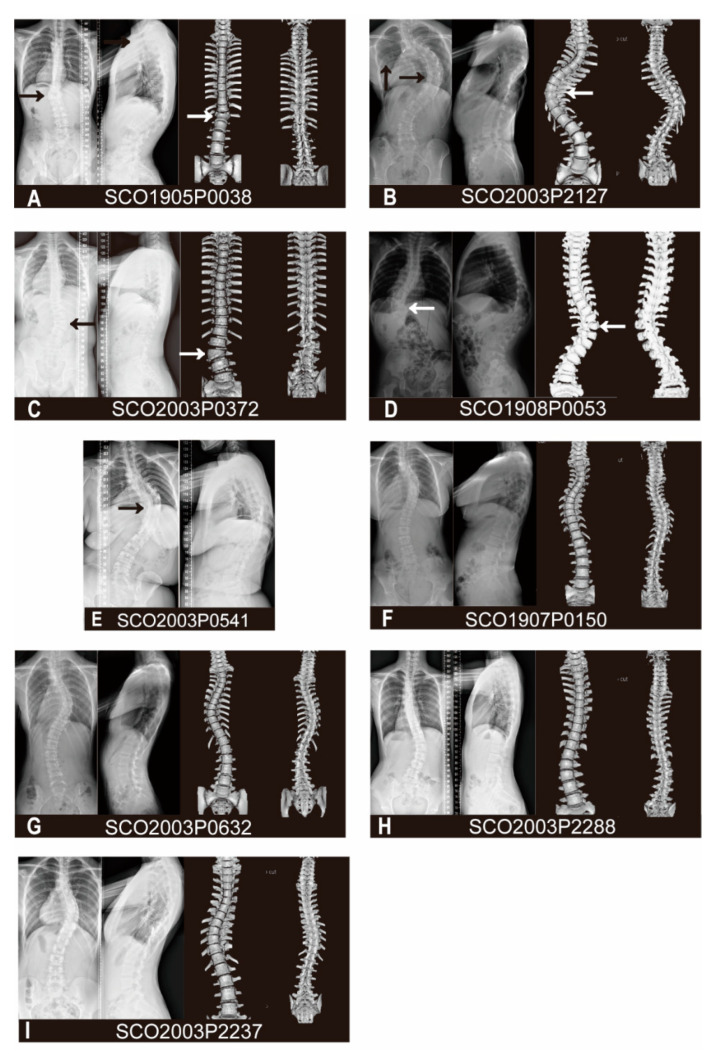
Antero-posterior, lateral spinal X-ray and the spinal three-dimensional CT reconstruction of patient SCO1905P0038 (**A**), SCO2003P2127 (**B**), SCO2003P0372 (**C**), SCO1908P0053 (**D**), SCO1907P0150 (**F**), SCO2003P0632 (**G**), SCO2003P2288 (**H**), SCO2003P2237 (**I**). Antero-posterior, lateral spinal X-ray of patient SCO2003P0541 (**E**). Arrowheads point to the vertebral or rib deformities.

Patient SCO1905P0038 is a 13-year-old male with T12 butterfly vertebra and T9-T10 segmentation defect. The spinal plain radiograph shows not only a coronal curve to the left, but also has a severe thoracolumbar kyphosis in the sagittal plane with a 65° Cobb angle, both results of continuous deformity in the vertebral body (Figure 2A). The patient has a heterozygous deletion between nucleotide 464 and 465 (c.464_465delAC, p.H155Pfs*16). This frameshift variant was mapped to the extracellular immunoglobulin region of PTK7 protein (Figure 1A) and is predicted to cause the early termination of mRNA translation. It is a novel variant, previously undescribed in mutational databases and is highly conserved across different vertebral species except zebrafish (Figure 1B).

Patient SCO2003P2127 is a 21-year-old female with a diagnosis of congenital scoliosis. This patient presents with the failure of segmentation in the concave side of T9-T11, fusion of the 4th and 5th ribs, and absence of 12th ribs. As a result, the patient has a severe imbalance of the spine in the coronal plane, with a 92° Cobb angle of the main curve at T5-L1 and a compensatory curve of 45° at the lumbar level (Figure 2B). She also has a history of patent ductus arteriosus. The heterozygous missense variant c.1394A>G (p.K465R) of the *PTK7* gene is mapped to the extracellular immunoglobulin region of the PTK7 protein (Figure 1A). This variant is previously unreported in mutational databases and is highly conserved (Figure 1B). It is predicted by CADD and PolyPhen-2 to be deleterious.

Patient SCO2003P0372 is an 8-year-old female with L2 hemivertebrae and L2-L3 segmentation defect. Her spine has a 38° right curve at the lumbar region with the hemivertebrae as its apex in the coronal plane, and a slight compensatory curve at the thoracic region (Figure 2C). She has a heterozygous missense variant c.1879G>A (p.G627R) of *PTK7*. This variant is located in the intracellular domain and adjacent to the transmembrane region of the PTK7 protein (Figure 1A). This variant is highly conserved (Figure 1B) and has been reported in the gnomAD database with low frequency. It is predicted by CADD and PolyPhen-2 to be deleterious.

Patient SCO1908P0053 is a 1-year-old female detected segmented wedge vertebrae in T11 and T12. This child displays severe imbalance in both the coronal and sagittal planes, in which the left curve reached 78° with the deformed vertebral body as the apex in the coronal plane, and severe kyphosis of 48° in the thoracolumbar region in the sagittal plane (Figure 2D). Due to her young age, long segment involvement, and the combination of thoracolumbar scoliosis and kyphosis, she received growth rod implantation and repeated growth rod extension processes over the past few years. Patient SCO2003P0541 is a 7-year-old female with segmentation failure from T7 to T11. Although the spinal deformity was discovered at the age of 7, the patient did not receive proper treatment until adulthood, leading to the continued progression of kyphoscoliosis. Similar to the patient SCO1908P0053, this patient has severe imbalance on both the coronal and sagittal planes in the thoracic spine, with a right curve of 87° and a thoracic kyphosis of 62°, which compared with the normal physiological thoracic kyphosis ranged from 10° to 40° (Figure 2E). She also suffered from chest deformity and diastematomyelia from MRI scans, which is consistant with the reported association between *PTK7* variants and neural tube defects (NTDs) [19]. The patient showed no dyspnea, sensory or motor disorders in the lower limbs, nor did she show urinal or excretory dysfunction. These two individuals carry the same heterozygous missense variant c. 1955G>T (p.R652L). This variant is located in the intracellular domain of PTK7, close to the pseudokinase (PK) domain (Figure 1A). This variant is conserved across vertebral species besides zebrafish (Figure 1B), and has been reported in gnomAD database with low frequency.

Patients SCO1907P0150, SCO2003P0632, SCO2003P2288 and SCO2003P2237 all suffer from adolescent idiopathic scoliosis (AIS). Patient SCO1907P0150 is an 11-year-old female. The patient has two curves on the coronal plane, a 47° left curve in the upper thoracic segment (T2-T6) and a 51° right curve in the thoracic segment (T6-T12) (Figure 2F). She had a congenital ventricular septal defect that was surgically treated. WES analysis reveals a missense variant c. 49C>T (p.L17F) in *PTK7*, which has not been reported in the mutational databases. This variant is located at the beginning of the extracellular portion of the PTK7 protein (Figure 1A). It is predicted by CADD and PolyPhen-2 score to be deleterious.

Patient SCO2003P0632 displays a 50° right curve and a 42° left curve at the thoracic and thoracolumbar levels, respectively, without significant trunk deviation (Figure 2G). A missense variant c. 353C>T (p.S118F) is identified in patient SCO2003P0632, a 12-year-old girl, which is also absent from mutational databases. It is predicted to be located at the junction between the first and second Ig domains of the extracellular part of PTK7 protein (Figure 1A). This variant is predicted by CADD and Polyphen-2 scores to be deleterious.

Patient SCO2003P2288 is a 14-year-old female with a c. 2290G>A (p.D764N) variant in *PTK7*. X-rays showed that the main curve was a 46° right-sided lumbar scoliosis, with a long compensatory curve of 25° at T1-T11 to maintain basic balance of the trunk (Figure 2H). This missense variant has been reported in gnomAD database with low frequency and the altered amino acid is highly conserved in vertebrates except zebrafish (Figure 1B). The variant is mapped to the PK domain of the intracellular portion of the PTK7 protein (Figure 1A). The pathogenicity assessment has contradictory results using CADD and Polyphen-2 scores.

Patient SCO2003P2237 is a 13-year-old girl. On the coronal plane, the patient presents a single thoracic curve from T7 to T12 with a Cobb Angle of 52°. Due to the severe vertebral rotation, a relatively obvious razor-back deformity is seen on the sagittal plane (Figure 2I). WES analysis reveals that she has a missense variant c.2384G>A (p.R795H) in *PTK7*. The variant amino acid is highly conserved in vertebrates (Figure 1B) and mapped to the PK domain of the intracellular section of the PTK7 protein (Figure 1A). Reported in gnomAD database with low frequency, it is predicted by both CADD and Polyphen-2 scores to be deleterious.

### 3.2. Western Blot and Immunocytochemistry Analyses

**Figure 3:**
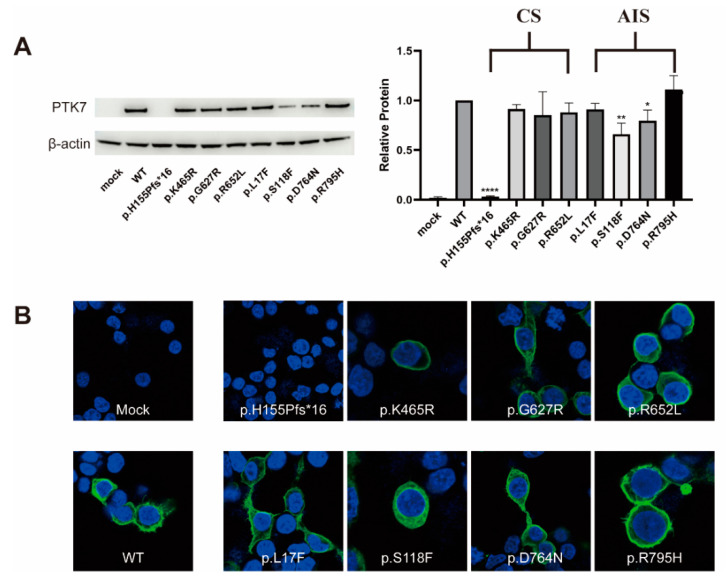
Western blot and ICC analyses. (**A**) HEK293T cells transiently transfected with WT or variant *PTK7* constructs revealed diminished PTK7 protein expression levels of p.H155Pfs*16, p.S118F and p.D764N. * *p* value < 0.05, ** *p* value < 0.01, **** *p* value < 0.0001. (**B**) ICC assays showed a faint signal of the frameshift variant PTK7 protein. No difference in PTK7 protein sub-cellular location was detected.

To identify the influence of the identified variants on PTK7 protein function, we evaluated overall protein expression and sub-cellular location of the variants of PTK7 compared to the WT. Western blotting analysis identified the immunoreactive-specific band for flag-tagged WT PTK7 at 150kDa and β-actin at 42kDa. The overall expression level was quantified by estimating bands from PNGase-treated samples and normalizing to WT. As anticipated, the overall expression of the frameshift variant (c.464_465delAC, p.H155Pfs*16) was significantly decreased compared to that of WT (*p* < 0.0001) (Figure 3A), indicating the loss-of-function effect of this variant. The expression level of two missense variants (p.S118F and p.D764N) were partially reduced (*p* = 0.0061 and *p* = 0.0293, respectively) (Figure 3A). Interestingly, these two variants were both identified in patients with AIS but not CS. There were no significant differences in the overall protein expression of the other missense variants (p.K465R, p.G627R, p.R562L, p.L17F, p.R795H) compared with the WT.

We also performed immunocytochemistry (ICC) assays and found there was a faintest signal from the frameshift variant of the PTK7 protein (Figure 3B), which supported the results of Western blotting assays. However, the sub-cellular location of all missense variant PTK7 protein did not change compared to WT (Figure 3B).

## 4. Discussion

Here, we performed WES on the genomes of 885 scoliosis patients, including 583 CS patients and 302 AIS patients, and identified seven missense variants and one frameshift variant in *PTK7*. Loss-of-function of PTK7 resulted in skeletal phenotypes in both zebrafish and mice. In zebrafish models, embryos with the *PTK7^hsc9^* variant (a mutant allele harboring a 10bp deletion that results in a frameshift and the incorporation of multiple premature termination codons) had defects in the convergence of both neuroectoderm and axial mesoderm tissues, and abnormal three-dimensional spinal curvature with growth and development [2]. In 2016, Grimes et al. reported that mutated *ptk7* zebrafish exhibited spinal curvature as well as hydrocephalus, ependymal cell (EC) ciliary dysfunction and abnormal rate and pattern of the cerebrospinal fluid (CSF) flow [20]. The *chuzhoi* mutant mice embryo showed several congenital abnormalities including neural tube, heart and lung defects caused by the disruption of PTK7 protein expression [21]. In human, LoF mutations in *PTK7* and the double-heterozygous mutation of *PTK7* and *VANGL2* were associated with spina bifida and increased the genetic risk of NTDs [19]. However, only one case of scoliosis with a *PTK7* mutation has been previously reported [11], and our identification of these eight variants expands the variant and phenotypic spectrum of *PTK7*.

The PTK7 protein consists of an extracellular continuous immunoglobulin domain, a transmembrane domain and an intracellular PK domain [1]. PTK7 interacts with several molecules in both canonical and non-canonical Wnt signaling as a co-receptor. These molecules include Wnt ligands, Wnt receptors as well as intracellular components such as Dvls and β-catenin [22]. According previous studies, the extracellular domains and intracellular domains of PTK7 play distinct roles. By constructing and studying *PTK7* with deletion of different domains, Hayes et al. found that the plasma membrane-tethered *Ptk7* extracellular domain (*Ptk7*ΔICD) was sufficient to promote normal PCP as well as the inhibition of canonical Wnt signaling [2], while the intracellular domains may play a specific role in oriented cell division, radial intercalation and cilia orientation [23,24,25,26]. The intracellular PK domain of PTK7 may act as a scaffold promoting the binding of intracellular proteins and other receptors. In *Xenopus*, the ptk7 intracellular domain was stimulated by Wnt5A and induced its translocation into the nucleus, which promoted cell and tissue movements [27]. Additionally, the C-terminal PTK7 species up-regulated cadherin-11 expressed in the mesoderm-derived tissues, and which regulates osteogenesis. It is possible that PTK7 and Cadherin-11 might interact in embryogenesis and regulate similar developmental processes [28,29]. In our study, the frameshift variant and three missense variants (p.L17F, p.S118F, and p.K465R) were located in the extracellular domains of the PTK7 protein, while the other four missense variants (p.G627R, p.R652L, p.D764N, and p.R795H) were mapped to the intracellular portion, with p.D764N and p.R795H variants being located in the pseudokinase domain. In our expression assay, one variant in the extracellular domain and one variant in the intracellular domain were shown to alter the expression of *PTK7*, suggesting that both domains are critical for the integrity of PTK7 protein.

Interestingly, the frameshift variant that caused almost no expression of PTK7 was found in the CS patient, while the two missense variants that resulted in the significant decreases in protein levels were both found in patients with AIS. The patient carrying the truncating variant was diagnosed to have a mixed subtype of CS, with failure of formation (T12 butterfly vertebra) and segmental disorder (T9-T10) and no other developmental malformations. The two patients with the missense variants (p.S118F and p.D764N) did not have congenital developmental deformities of the vertebral body or other systems, presenting simple thoracolumbar scoliosis. However, due to the complex etiology of AIS, the role of PTK7 in AIS still warrants further validation and investigation.

Taken together, we hypothesize that the pathogenesis of vertebral deformities and the onset time of spinal curvature may be due to the effect of *PTK7* variants on the protein expression. In other words, congenital and adolescent idiopathic scoliosis may have a common genetic basis. Although some missense variants in our study did not show abnormalities in protein expression or sub-cellular location, it is possible that the altered amino acids can affect the structure of the protein and subsequently affect downstream signaling pathways. Therefore, it is necessary to further explore the possible downstream pathways of PTK7, such as the canonical and non-canonical Wnt pathways.

## 5. Conclusions

In conclusion, we identified eight *PTK7* variants in our mixed scoliosis cohort, including one frameshift variant (c.464_465delAC) and seven missense variants (c.49C>T, c.353C>T, c.1394A>G, c.1879G>A, c. 1955G>T, c.2290G>A, c.2384G>A). The frameshift variant resulted in a depleted expression of PTK7 protein, and two of missense variants caused reduced expression of PTK7. Our study extended the variant and phenotype spectrums of *PTK7* and suggested a common genetic basis of CS and AIS.

## Figures and Tables

**Table 1:** Summary of clinical features of the *PTK7* variant carriers.

Patient Number	Variant	Gender	Age of Onset	Diagnosis	Vertebral Malformations	Other skeletal Malformations or Deformities	Cardiac Abnormalities	Spinal Canal Deformities	Visceral Abnormalities
SCO1905P0038	c.464_465delAC	M	13	CS	T12 butterfly vertebra, T9-T10 failure of segmentation	None	None	None	None
SCO2003P2127	c.1394A>G	F	21	CS	None	Fusion of 4th and 5th ribs, 12th ribs absent	Postoperative of patent ductus arteriosus	None	None
SCO2003P0372	c.1879G>A	F	8	CS	L2 Hemivertebra, L2-L3 fusion	None	None	None	None
SCO1908P0053	c.1955G>T	F	1	CS	T11, T12 segmented wedge vertebrae	None	None	None	None
SCO2003P0541	c.1955G>T	F	7	CS	T7-T11 failure of segmentation	Chest deformity	None	Diastematomyelia	None
SCO1907P0150	c.49C>T	F	11	AIS	None	None	Ventricular septal defect	None	None
SCO2003P0632	c.353C>T	F	12	AIS	None	None	None	None	None
SCO2003P2288	c.2290G>A	F	14	AIS	None	None	None	None	None
SCO2003P2237	c.2384G>A	F	13	AIS	None	None	None	None	None

M, male; F, female; CS, congenital scoliosis; AIS, adolescent idiopathic scoliosis.

**Table 2:** Sequence variants identified in *PTK7*.

Patient Number	cDNA Change	Protein Change	Variant Type	Position	Gnomad_Exome_ALL	Gnomad_Exome_EAS	Gnomad_Genome_ALL	Gnomad_Genome_EAS	CADD	Ployphen-2 HDV Score	Evidence of Pathogenicity by ACMG	ACMG Classification
SCO1905P0038	c.464_465delAC	p.H155Pfs*16	Frameshift	43097560	0	0	0	0	NA	NA	PVS1+PM2+PP3	Pathogenic
SCO2003P2127	c.1394A>G	p.K465R	Missense	43109684	0	0	0.00003247	0.0006	26.2	0.997	PP3	Uncertain significance
SCO2003P0372	c.1879G>A	p.G627R	Missense	43112206	0.00004873	0.0006	0.00003232	0.0006	26.9	0.986	PP3	Uncertain significance
SCO1908P0053 SCO2003P0541	c.1955G>T	p.R652L	Missense	43112282	0.00007718	0.0005	0	0	23.6	0.947	PP3	Uncertain significance
SCO1907P0150	c.49C>T	p.L17F	Missense	43044275	0	0	0	0	19.95	0.997	PM2	Uncertain significance
SCO2003P0632	c.353C>T	p.S118F	Missense	43096988	0	0	0	0	25.6	0.982	PM2	Uncertain significance
SCO2003P2288	c.2290G>A	p.D764N	Missense	43114395	0.00000814	0.000058	0	0	23.8	0.114	PM1+PP3	Uncertain significance
SCO2003P2237	c.2384G>A	p.R795H	Missense	43126607	0.00000406	0	0.00003231	0	30	0.986	PM1+ +PP3	Uncertain significance

The RefSeq transcript sequence used for PTK7 is NM_152881.3. Abbreviations: NA, not available. ACMG, American College of Medical Genetics and Genomics. PVS, pathogenic very strong. PM, pathogenic moderate. PP, supporting pathogenicity.

## Data Availability

Data are available upon reasonable request. The datasets analyzed during the current study are available from the corresponding author on reasonable request.
